# Heterogeneous abnormalities of *in-vivo* left ventricular calcium influx and function in mouse models of muscular dystrophy cardiomyopathy

**DOI:** 10.1186/1532-429X-15-4

**Published:** 2013-01-16

**Authors:** Elizabeth Greally, Benjamin J Davison, Alison Blain, Steve Laval, Andrew Blamire, Volker Straub, Guy A MacGowan

**Affiliations:** 1 Institute of Genetic Medicine, Newcastle University, International Center for Life, Newcastle, UK; 2 MR Centre, Newcastle University, Newcastle, UK; 3 Dept of Cardiology, Freeman Hospital and Newcastle University, Newcastle upon Tyne, NE7 7DN, UK

**Keywords:** Muscular dystrophy, Cardiomyopathy, Magnetic resonance imaging, Calcium

## Abstract

**Background:**

Manganese-enhanced cardiovascular magnetic resonance (MECMR) can non-invasively assess myocardial calcium influx, and calcium levels are known to be elevated in muscular dystrophy cardiomyopathy based on cellular studies.

**Methods:**

Left ventricular functional studies and MECMR were performed in *mdx* mice (model of Duchenne Muscular Dystrophy, 24 and 40 weeks) and *Sgcd−/−* mice (Limb Girdle Muscular Dystrophy 2 F, 16 and 32 weeks), compared to wild type controls (C57Bl/10, WT).

**Results:**

Both models had left ventricular hypertrophy at the later age compared to WT, though the *mdx* mice had reduced stroke volumes and the *Sgcd−/−* mice increased heart rate and cardiac index. Especially at the younger ages, MECMR was significantly elevated in both models (both *P*<0.05 versus WT). The L-type calcium channel inhibitor diltiazem (5 mg/kg i.p.) significantly reduced MECMR in the *mdx* mice (*P*<0.01), though only with a higher dose (10 mg/kg i.p.) in the *Sgcd−/−* mice (*P*<0.05). As the *Sgcd−/−* mice had increased heart rates, to determine the role of heart rate in MECMR we studied the hyperpolarization-activated cyclic nucleotide-gated channel inhibitor ZD 7288 which selectively reduces heart rate. This reduced heart rate and MECMR in all mouse groups. However, when looking at the time course of reduction of MECMR in the *Sgcd−/−* mice at up to 5 minutes of the manganese infusion when heart rates were matched to the WT mice, MECMR was still significantly elevated in the *Sgcd−/−* mice (*P*<0.01) indicating that heart rate alone could not account for all the increased MECMR.

**Conclusions:**

Despite both mouse models exhibiting increased *in-vivo* calcium influx at an early stage in the development of the cardiomyopathy before left ventricular hypertrophy, there are distinct phenotypical differences between the 2 models in terms of heart rates, hemodynamics and responses to calcium channel inhibitors.

## Background

Muscular dystrophies are frequently associated with cardiomyopathies. In Duchenne muscular dystrophy (DMD), which is caused by the absence of dystrophin, either clinical or subclinical dilated cardiomyopathy is invariably present [1,2]. Recessive mutations in one of the genes for α-, β-, γ- or δ-sarcoglycan cause a heterogeneous group of autosomal recessive limb girdle muscular dystrophies (LGMD2C-F), and these also frequently have dilated cardiomyopathy, especially those patients with LGMD2F caused by mutations in the δ-sarcoglycan gene [3].

A hypothesis to explain the underlying pathophysiology of cardiomyopathies associated with muscular dystrophy is that recurrent membrane injury leads to an increased influx of calcium [4] which then causes downstream effects such as activation of calcium-dependent hypertrophic pathways [5], reactive oxygen species [6] and cell death through necrosis with mitochondrial defects [7]. Membrane injury may not be the only mechanism whereby calcium enters cardiomyocytes. Cohn et al. [8] have shown that the L-type calcium channel antagonist verapamil restores left ventricular function in *Sgcd−/−* mice, though has no effects in *mdx* mice. Whereas this was explained at that time on the basis of effects on coronary vasculature, subsequent studies have suggested that this was a direct cardiomyocyte effect [7,9]. That there are differences between the *Sgcd−/−* and *mdx* mice has also recently been highlighted by a study showing marked hemodynamic differences in response to β-blockers [10].

Manganese-enhanced cardiovascular magnetic resonance (MECMR) uses 2 properties of the manganese ion to allow *in-vivo* assessment of calcium influx [11]. Manganese enters cardiomyocytes through calcium channels, and is also a T_1_ contrast agent with CMR. Thus, increased calcium influx with dobutamine increases contrast-enhancement, and decreased influx with the calcium channel blocker diltiazem reduces contrast. We used MECMR to test the hypothesis that *in-vivo* calcium influx in muscular dystrophy cardiomyopathy mice is increased at an early stage in the development of the cardiomyopathy and so is an important factor in disease progression. To test this hypothesis we studied *in-vivo* left ventricular function, and MECMR in *Sgcd−/−* and *mdx* mice, determining the roles of ageing, L-type calcium channel inhibition and heart rate in the altered manganese contrast enhancement pattern of these models.

## Methods

Animals: Two separate age groups of male mice were used in this study. C57BL/10ScSnOlaHsd (wild type, WT, Harlan Laboratories, Indianapolis, USA) and *mdx* mice (C57BL/10ScSn-mdx/J, Jackson, Maine USA) were scanned at ages 24 and 40 weeks, and *Sgcd−/−* mice were scanned at 16 and 32 weeks (on C57Bl/6 background, a kind gift from Dr Kevin Campbell, University of Iowa). These ages were chosen based on previous data reflecting the earlier development of the cardiomyopathy in the *Sgcd−/−* compared to the *mdx* mice [12,13]. Numbers of mice in the various experiments are detailed in the table and figure legends. The investigation conforms with Directive 2010/63/EU of the European Parliament and was performed under the terms of the Animals (Scientific Procedures) Act 1986, authorized by the Home Secretary, Home Office UK. The *Sgcd−/−* mice were compared with the 8 week older C57/BL10 mice as controls, as preliminary data showed no differences in any measure of left ventricular function or manganese uptake between 16 and 24 week old C57/BL10 mice (data not shown).

### CMR

Mice were anaesthetized using 5% isofluorane and anaesthesia maintained at 1.5% in oxygen with a flow rate of 0.5 L/min. The tail-vein was cannulated and mice placed on a MR compatible sled with surface ECG electrodes on the chest wall, respiration pillow and cutaneous temperature probe (Dazai Research Instruments, MICe, Toronto, Canada). This was connected to MR compatible monitoring equipment (SA Instruments Inc., Stony Brook, NY 11790). Mice on the sled were then placed on a bed holding the nose cone for anaesthesia delivery and then all of this slid into a 39 mm diameter quadrature birdcage volume coil (Rapid Biomedical GmbH). Images were acquired on a 7 Tesla horizontal bore microimaging system equipped with a 12-cm microimaging gradient insert (maximum gradient 40 gauss/cm) (Varian Inc., Palo Alto, CA, USA). Following power calibration and global shimming a series of four pilot transverse images were acquired over the heart. Single slice coronal and sagittal images were then obtained in order to view the apex and mitral valve planes. These images were used to plan for the true short axis plane [14]. To measure left ventricular function, contiguous short axis slices were acquired to cover the entire left ventricle using a spoiled gradient-echo cine sequence (TR=5 ms, TE=1.42 ms, flip angle 15°, FOV 30×30 mm, data matrix 128×128, 1 mm slice thickness, max. of 10 slices depending on heart size, 4 averages). Images were ECG triggered to the R wave with a cine delay of 15 ms and typically 30 phases were acquired distributed through the cardiac cycle (depending on HR). Images were zero-filled to a matrix size of 256×256. Scans were converted to matfiles (using a matlab script kindly provided by Dr Johannes Riegler, University College London, UK) and analysed using the freely available analysis software Segment v1.8 (http://segment.heiberg.se) [15] to measure left ventricular mass and the LV functional parameters – end-systolic and end-diastolic volumes, stroke volume, ejection fraction and cardiac output.

### Manganese enhanced CMR (MECMR)

**Figure 1:**
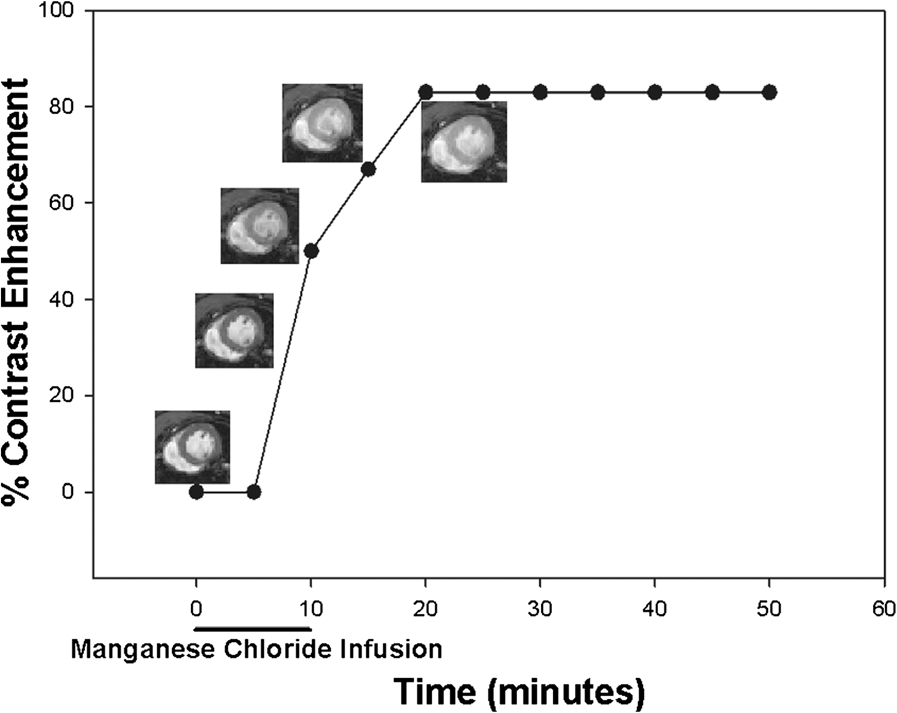
Representative figure showing single example of manganese contrast enhancement experiment, with superimposed images showing clear increase in myocardial contrast following manganese infusion.

For the manganese contrast enhancement, 60 mM manganese chloride (Sigma-Aldrich 244589) was given by intravenous infusion through the tail vein cannula at a flow-rate of 0.6 ml/hour, flow time adjusted according to weight to give a total dose of 190 nmol/g body weight. For a 30 g mouse, this would result in a 9.5 minute infusion. Gradient echo short axis images at the level of the papillary muscles (T_1 _weighted parameters: TR=35 ms, TE=3.5 ms, flip angle 60°, FOV 30 mm × 30 mm, data matrix 128 × 128, 1 mm slice thickness, 6 averages) were taken. Four baseline images were acquired in order to average any variations due to changes in TR as a result of fluctuations in heart rate and then at 5 minute intervals for 50 minutes during and after manganese infusion. A relative increase in T_1 _weighted contrast indicates increased manganese uptake (Figure 1). Images acquired from each timepoint were extracted using a Python script (kindly written by Dr Conor Lawless, Newcastle University), opened in Image J (http://rsb.info.nih.gov/ij/) and converted to a stack using the stack builder plugin. On the first image an area of interest was drawn to fit inside the myocardium and average signal intensity measured. Minor adjustments of this drawn region were made for subsequent images in the stack and the increase in myocardial contrast enhancement expressed as a percentage increase from the average of the four baseline images (which showed little or no variation).

### Pharmaceutical modification of manganese contrast enhancement

The L-type calcium channel antagonist diltiazem (5–10 mg/kg body weight i.p., 10 mg/kg being maximal tolerated dose, Sigma Chemical Co., St Louis, MO) was given separately to subgroups of the younger mice. The hyperpolarization-activated cyclic nucleotide-gated (HCN) channel inhibitor ZD 7288 (0.3 mg/kg body weight i.p., Sigma Chemical Co., St Louis, MO) was also given to separate groups to study the effects of selective heart rate reduction on manganese contrast enhancement [16,17]. For the ZD 7288 experiments we also included a 2.5 minute scan as we were particularly interested in the early kinetics of manganese uptake related to heart rate. These medications were given prior to placing the animal in the scanner which was approximately 30–40 minutes before the manganese infusion.

### Statistics

To test effects of time and age on manganese-contrast enhancement, repeated measures of analysis of variance was used combining data from young and old groups. Within each age group, analysis of variance was used to test differences amongst groups comparing an average value of steady state manganese contrast enhancement, and MR functional data. Post-hoc testing was performed with the Scheffe′ test. Single comparisons were made with Student’s t-tests. Data are expressed as mean ± standard error of the mean.

## Results

### MR haemodynamics: Left ventricular hypertrophy in older *Sgcd−/−* and *mdx* mice, increased heart rates and cardiac index in *Sgcd−/−* mice, and reduced stroke volume in *mdx* mice

Age had significant effects on haemodynamics (ANOVA, all 3 groups combined), with significant increases in end-diastolic volume (*P*<0.01), end-systolic volume, and stroke volume (both *P*<0.05) (data not shown). As there were significant differences in body weights, left ventricular mass, volume measurements and cardiac output are indexed to body weight (i.e. cardiac index), and with that changes in volumes were no longer significant (Table 1). *Sgcd−/−* mice had lower body weights, higher heart rates, increased left ventricular mass index at the older age, normal volumes, and increased cardiac index at both ages. The increased cardiac index was a result of the higher heart rate, as stroke volume index was not significantly different to WT mice. The *mdx* mice had significant left ventricular hypertrophy in the older age group (though left ventricular mass index did not actually change in these mice with age, rather it was decreased in older WT mice), and reduced stroke volume index when considering both age groups combined compared to WT. The *mdx* mice also had significantly lower ejection fractions compared to the *Sgcd−/−* mice. Thus, there were markedly different haemodynamic characteristics in the 2 muscular dystrophy mice models. Whereas both had left ventricular hypertrophy at older ages when compared to older WT, there was a hyperdynamic circulation in the *Sgcd−/−* mice with increased cardiac index, and left ventricular systolic dysfunction with reduced stroke volume in the *mdx* mice. Average surface temperatures measured during the scans were not significantly different between the groups (WT: 34.3±0.3°C, *mdx* 33.5±0.4°C and *Sgcd−/−* 34.3±0.3°C (ANOVA p=NS).

**Table 1:** Haemodynamics in young and older mice

	***YOUNG***			***OLD***		
	**WT**	***Mdx***	***Sgcd−/−***	**WT**	***Mdx***	***Sgcd−/−***
**N**	11	10	10	7	9	13
**Body weight, g**	33.3±0.9*	35.8±1.0**	28.8±1.0	40.5±1.2**	37.1±1.1	32.9±0.9
**Heart rate, bpm**	390±13**	419±14	480±14	384±16**	427±15	483±12
**LV mass index, (×10**^**-3**^**)**	3.03±0.12	3.57±0.13	3.58±0.13	2.80±0.14**	3.56±0.13^+^	3.86±0.11
**EDV index, μl/g**	1.97±0.11	1.72±0.11	2.06±0.11	1.93±0.14	1.78±0.12	1.94±0.10
**ESV index, μl/g**	0.70±0.07	0.68±0.08	0.67±0.08	0.72±0.09	0.74±0.08	0.69±0.07
**Stroke volume index, μl/g**	1.28±0.06	1.03±0.06**/^†^	1.38±0.06	1.21±0.07	1.04±0.06^†^	1.25±0.05
**Ejection fraction, **%	65±2	61±2^††^	68±2	63±3	60±2^††^	65±2
**Cardiac index, ml/min/g**	0.50±0.03**	0.43±0.03**	0.67±0.03	0.46±0.03^#^	0.44±0.03**	0.60±0.03

LV: left ventricle; EDV: end-diastolic volume; ESV, end-systolic volume.

*P<0.01 vs older WT.

**P<0.01 vs *Sgcd−/−* within same age group.

^+^*P*<0.05 vs WT within same age group.

^#^*P*=0.06 vs *Sgcd−/−* within same age group.

^†^*P*<0.01 ANOVA vs WT and *Sgcd−/−* both ages combined.

^††^*P*<0.05 ANOVA vs *Sgcd−/−* both ages combined.

### MECMR: Slower kinetics of manganese uptake in all older mice, and increased contrast enhancement in younger *Sgcd−/−* and *mdx* mice

**Figure 2:**
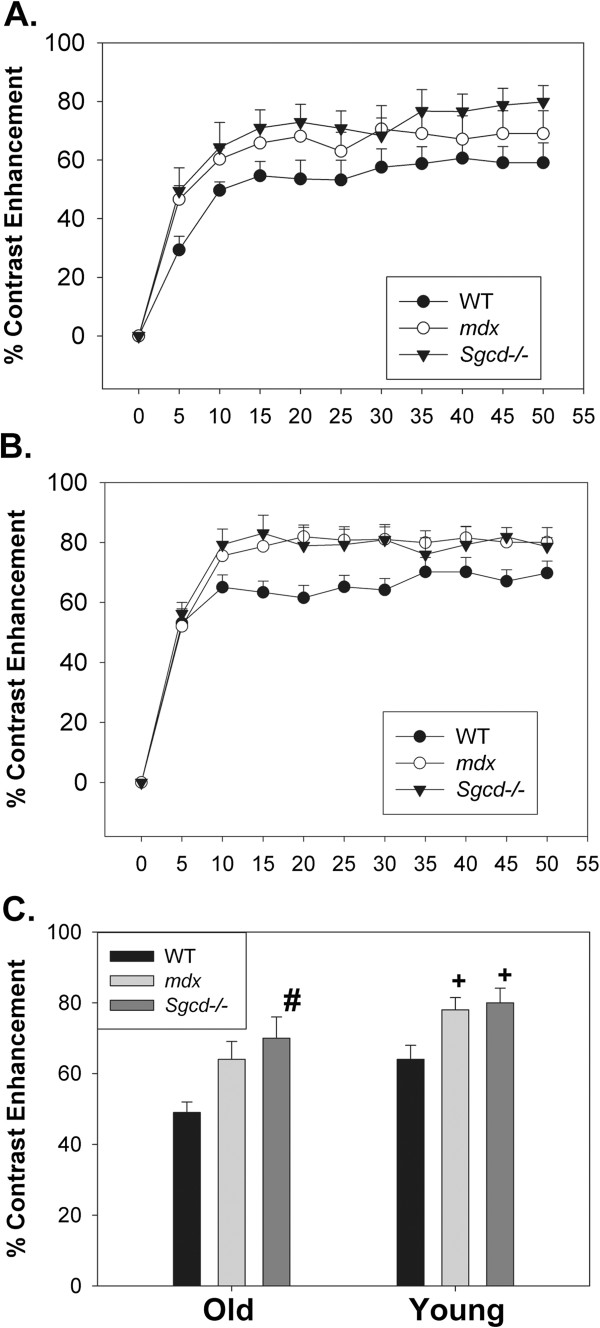
**Effects of Ageing on Manganese Contrast Enhancement.** Manganese contrast enhancement in older mice (**A**) and young mice (**B**). The kinetics of manganese contrast enhancement are slower in the older mice compared to younger mice (*P*<0.05 repeated measures ANOVA). **C**. Mid point contrast enhancement in older and younger mice. There was a borderline increase in contrast enhancement in the older *Sgcd−/−* mice (^#^*P*=0.07 versus WT) and in the younger mice contrast enhancement was significantly elevated in both *Sgcd−/−* and *mdx* mice (^+^*P<*0.05 versus WT). For the younger mice N as are in Table 1, in the older mice WT N = 9, *mdx* N = 10, and *Sgcd−/−* mice N = 15 (as some older mice did not have functional data).

Contrast-enhancement was significantly slower in all older mouse groups compared to younger mice (Figures 2A and B, P<0.05, time*age interaction, repeated measures ANOVA combining both age groups and all 3 groups). For instance, in older WT mice contrast-enhancement at 5 minutes was 29±5% compared with 53±5% in younger WT mice. This analysis also showed that when considering both ages together contrast enhancement was significantly elevated in the *Sgcd−/−* mice relative to controls (*P*<0.05 post hoc Scheffe test), and this was of borderline significance for the *mdx* mice (*P*=0.08). To study contrast-enhancement within the 2 age groups, a mid point value of contrast enhancement was defined as the mean contrast enhancement of the steady state period of 10 to 25 minutes after start of the manganese infusion, with 5 minute interval scans. In the older mice, mid point contrast enhancement was borderline increased in the *Sgcd−/−* compared to WT mice (P=0.07, post hoc Scheffe test), and not significantly different in the *mdx* mice (Figure 2C). In the younger mice, mid point contrast enhancement was significantly increased in both the *Sgcd−/−* and *mdx* compared to WT mice (both *P*<0.05, post hoc Scheffe test). As the *Sgcd−/−* mice were on a different strain background to the C57Bl/10 controls we performed initial experiments to determine if there was a strain difference between C57Bl/10 and C57Bl/6 mice. In these experiments the mid point contrast enhancement was 50±6% in C57Bl/10 (N=5) and 50±10.0% in C57Bl/6 mice (N=3, p=NS) indicating that there was no strain effect.

### Pharmaceutical modification of calcium channels: Resistance to L-type calcium channel blockade in young *Sgcd−/−* mice

**Figure 3:**
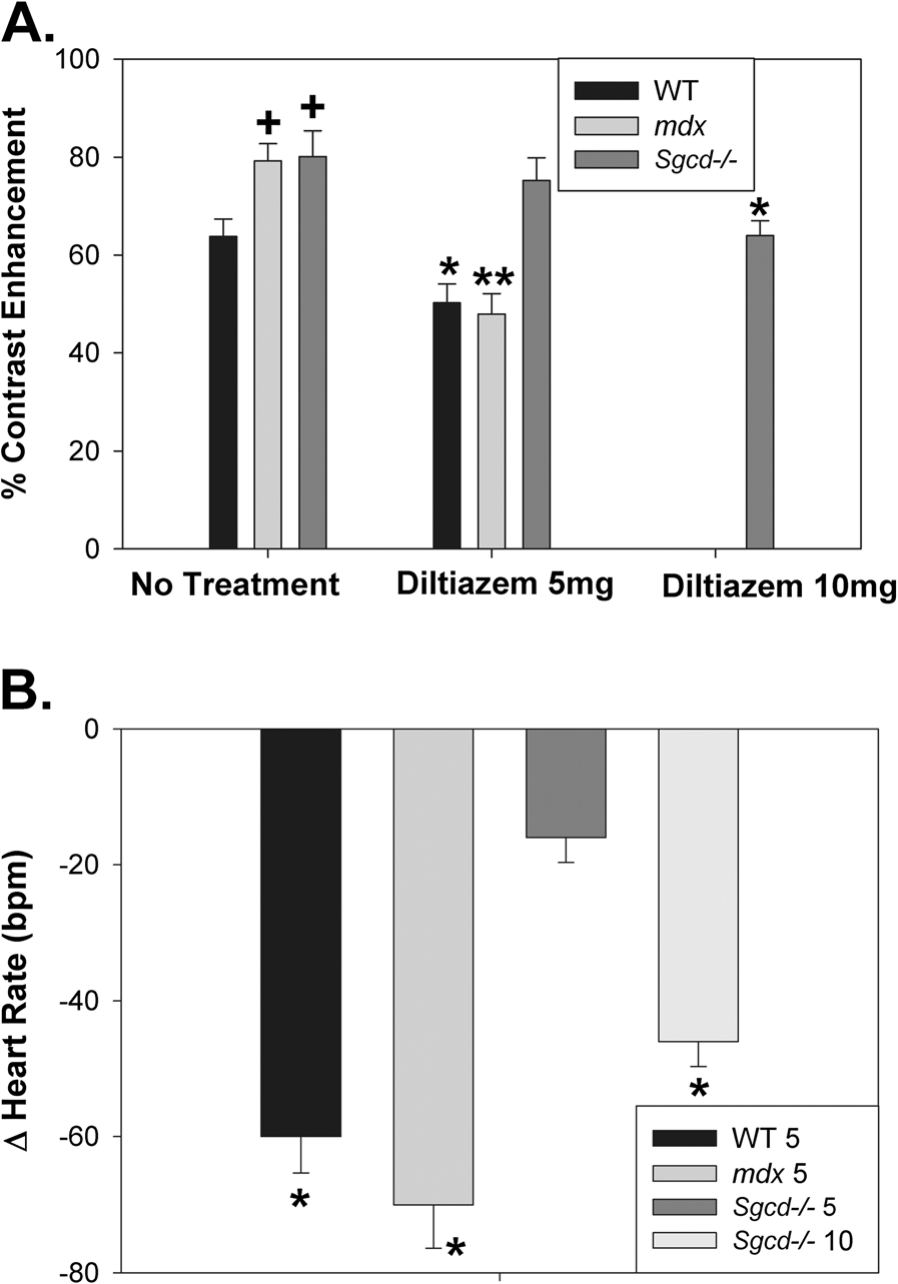
**Effects of L-type Calcium Channel Blocker Diltiazem on Manganese Contrast Enhancement. A**. Mid point contrast enhancement in younger mice with no pharmacological treatment (left column), showing significant higher levels of mid point contrast enhancement in *mdx* and *Sgcd−/−* vs WT mice (^+^*P<*0.05 versus WT, as in Figure 2C). Diltiazem 5 mg produced significant reductions in contrast enhancement in *mdx* (N=7) and WT (N=6) versus no treatment, though not for the *Sgcd−/−* mice (N=6). Diltiazem 10 mg produced a significant reduction in contrast enhancement in the *Sgcd−/−* mice (N=9); (^+^P<0.05 versus WT; *P<0.05 and **P<0.01 versus untreated, within same strain). **B**. Effects of diltiazem on heart rates. Diltiazem (5 mg) significantly reduced heart rates in WT and in *mdx* mice, though not in *Sgcd−/−* mice, a similar effect as seen with manganese contrast enhancement. Higher doses of diltiazem (10 mg) significantly reduced heart rates in the *Sgcd−/−* mice. (*P<0.05 versus without diltiazem).

We concentrated on the younger mice for the pharmaceutical studies as these were the ages where the most significant increases in contrast enhancement were seen. The L-type calcium channel blocker diltiazem (5 mg/kg i.p.) significantly reduced contrast enhancement (Figure 3A) in WT (21%, P<0.05) and to a greater extent in *mdx* mice (38%, P<0.001), though had no significant effects in the *Sgcd*−/− mice (6%, P=NS). A higher dose of diltiazem (10 mg/kg) did produce significant reductions in contrast enhancement in *Sgcd−/−* mice (21%, *P*<0.05), indicating that the L-type calcium channels were partially resistant in the *Sgcd−/−* mice. Consistent with the variable sensitivity to L-type calcium channel blockade, diltiazem 5 mg/kg significantly reduced heart rates in the WT and *mdx* mice, but not in the *Sgcd−/−* mice, though 10 mg/kg did produce significant reductions in the *Sgcd−/−* mice (Figure 3B).

### The role of heart rate in manganese contrast enhancement

**Figure 4:**
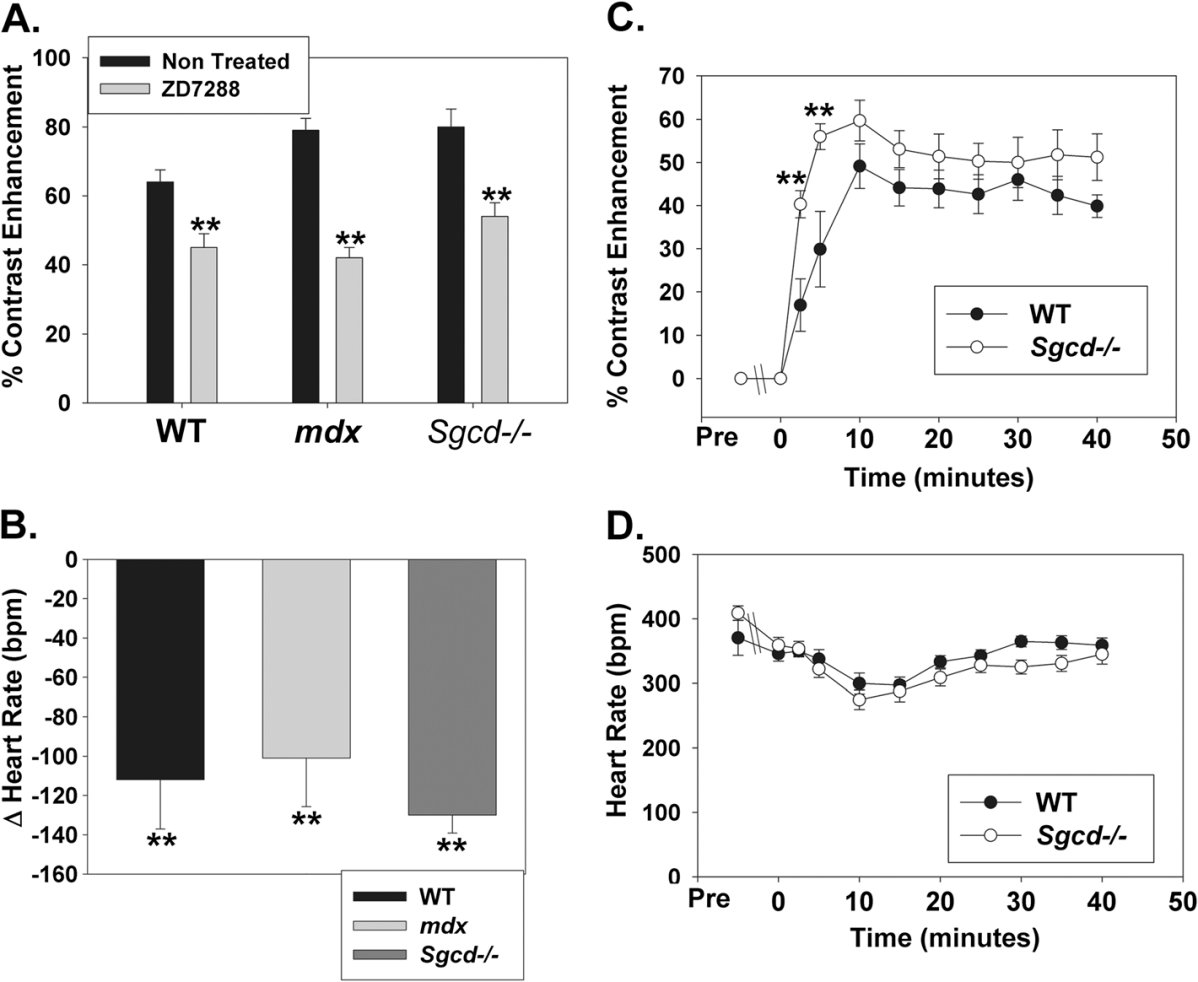
**Effect of Heart Rate Reduction on Manganese Contrast Enhancement. A** and **B**. The HCN channel inhibitor ZD 7288 significantly reduced mid point contrast enhancement in all mouse groups (**P<0.01 versus without ZD 7288), and this was accompanied by significant reductions in heart rate also in all groups (**P<0.01 versus heart rate pre ZD 7288) (WT N = 8, *mdx* N = 9, *Sgcd−/−* N = 10). **C** and **D**. When the time course of manganese contrast enhancement and heart rate in the wild type and *Sgcd−/−* mice is examined we can see that the ZD 7288 reduces heart rate in the *Sgcd−/−* mice to the same level as wild type mice from the time of starting the manganese infusion, though during the first 5 minutes there is still a significantly higher level of manganese contrast enhancement in the *Sgcd−/−* mice compared to WT (**P<0.01). This indicates that whereas heart rate is an important factor in the extent of manganese contrast enhancement, in the *Sgcd−/−* mice there is at least some component of increased cellular uptake of manganese per heart beat compared to WT.

There was increased contrast-enhancement in the *Sgcd−/−* mice which accompanied a significantly higher heart rate compared to WT mice (Table 1), and thus the mechanism of this increased contrast-enhancement could be due to more heart beats and action potentials transmitting Mn^2+^ into the cell, though could also be related to a cellular channel defect with increased Mn^2+^ entry into the myocyte per action potential. To clarify this issue we used the HCN channel inhibitor ZD 7288 to selectively reduce heart rate in all 3 groups of younger mice. ZD 7288 significantly reduced contrast enhancement in all 3 groups of mice, and this was associated with significant reductions in heart rate (Figure 4A and B). This does suggest in general that the manganese contrast enhancement signal is sensitive to heart rate, and care must therefore be taken when comparing mouse models with different heart rates with this technique. However, when comparing the time plots of contrast enhancement and heart rates in the WT and *Sgcd−/−* mice (Figure 4C and D) we see that at a very early stage in the increase in contrast enhancement (2.5 and 5 minutes) when heart rates of both groups of mice are very similar that there was still a marked increase in contrast-enhancement in the *Sgcd−/−* mice compared to wild type. Thus, while contrast-enhancement is sensitive to heart rate, by reducing the heart rate in the *Sgcd−/−* mice to WT levels we can see that there is clearly a component of increased cellular uptake of Mn^2+ ^per action potential. The pattern of early increased contrast enhancement was not however seen in the *mdx* mice. For instance, at 2.5 minutes with similar heart rates to WT mice contrast enhancement was 22±5% in the *mdx* mice compared to 17±6% in the WT mice (p=NS), so this illustrates a different pattern to the *Sgcd−/−* mice, and potentially different mechanism of abnormal contrast enhancement.

## Discussion

In muscular dystrophy cardiomyopathy at an early stage in the development of the cardiomyopathy before significant left ventricular hypertrophy in both the *mdx* and *Sgcd−/−* mouse models, *in-vivo* calcium influx is increased (as determined by manganese contrast enhancement). However, there are clear differences in both left ventricular function and myocardial calcium influx between the 2 models. The *mdx* mouse has left ventricular systolic dysfunction with a reduction in stroke volume, whereas the *Sgcd−/−* mice have increased heart rates, cardiac index and so a hyperdynamic circulation. Ejection fractions are lower in the *mdx* compared to the *Sgcd−/−* mice. These are also differences in the responses to L-type calcium channel blockade in that there is a relative resistance in the *Sgcd−/−* mice though *mdx* mice exhibit a similar response to wild type mice. As heart rate is increased in the *Sgcd−/−* mice, this is potentially a cause of the increased contrast enhancement, and selective heart rate reduction reduces contrast enhancement in all groups. Nevertheless, it is clear in the *Sgcd−/−* mice that when heart rates are reduced to wild type levels, there is still increased manganese contrast enhancement indicating a primary cellular defect in calcium influx. This increase in early contrast enhancement after heart rate reduction is not seen with the *mdx* mice.

### L-type calcium channels in muscular dystrophy cardiomyopathy

We were interested in L-type calcium channel blockers for 2 reasons – 1) they can reduce manganese contrast enhancement [11], and 2) have differential effects in the *mdx* and *Sgcd−/−* mice [8]. It is known that intracellular calcium is increased in muscular dystrophy cardiomyopathy [7,18]. Cohn et al. [8] have shown that the L-type calcium channel blocker verapamil had significant beneficial effects in the *Sgcd−/−* mice on troponin I levels and cardiac histology, though not in the *mdx* mice. To reconcile the findings in this study and our findings it is necessary to examine doses used. The doses used in Cohn et al. compared to the present one are different when corresponding human doses calculated by weight are examined, though a consistent theme is that high doses are required. The doses of diltiazem in the current study of 5–10 mg/kg would equal 350–700 mg in a 70 kg human, the upper limit of a normal human dose being 240 mg. The dose of verapamil used by Cohn et al. was 3.5 mg/day, which would be approximately 35 fold higher than an appropriate human dose. One possible consistent explanation is that only higher doses of L-type calcium channel antagonists are effective in the *Sgcd−/−* mice, as we only show responses to higher doses of diltiazem. There are strain differences in the backgrounds of the wild type and *mdx* (both C57Bl/10) versus the *Sgcd−/−* mice (C57Bl/6). Though we could find no differences between Bl/10 and Bl/6 mice in terms of manganese uptake, we cannot rule out some strain effect in the response to L-type calcium channel blockade.

### Role of heart rate in manganese contrast enhancement

When comparing models with different heart rates, it may be difficult to determine whether a difference in manganese contrast enhancement is due to the increased heart rate resulting in more action potentials over the period of the manganese infusion or whether there is an underlying cellular defect with an altered calcium influx per action potential [19]. To determine whether the increased heart rate in the *Sgcd−/−* mice accounts for the increased manganese contrast enhancement we have used the HCN channel inhibitor ZD 7288 which inhibits the I_*f *_channel producing a selective reduction in heart rate [16,17]. These studies clearly show that at matched heart rates, the *Sgcd−/−* mice have increased manganese contrast enhancement compared to wild type, indicating a primary cellular defect in calcium influx. These experiments also show clearly the important role that heart rate plays in manganese contrast enhancement, so that for instance, in our experiments with diltiazem it is reasonable to assume that some of the reduction in contrast enhancement is due to a reduction in heart rate. Of note, a small amount of calcium may enter cardiac myocytes through the I_*f *_channel (0.5% of the current from the HCN2 channel) [20], though this small amount is unlikely to have significantly effected our results. Furthermore, as the *mdx* mice do not have significantly different heart rates compared to wild type mice the increase in manganese contrast enhancement will be due to a primary cellular defect in calcium influx. Surface temperatures of the mice were below physiological levels during the scans, and so heart rates were also low. Surface temperatures are approximately 1°C lower than core body temperature [21]. Higher temperatures would have likely resulted in higher heart rates and this may alter manganese uptake rates.

### Age-related changes in myocardial calcium influx

Isenberg et al. [22] have shown that in aged mice calcium transients peak late and decay slowly. Salameh et al. [23] have shown that the L-type calcium (I_Ca.L_) current is abnormal in aging rabbit hearts with the I_Ca.L _density normalised to cell volume significantly reduced, maximum conductance also significantly decreased and steady state inactivation shifted to more positive potentials in aged hearts. However, expression of the α_1c _subunit of the L-type channel, the SERCA2a-ATPase, and the Na^+^/Ca^2+ ^exchanger did not differ significantly between the two age groups. Direct comparison of cellular calcium handling and MECMR would be very informative in studying age-related changes in calcium influx. Our young and old mice were separate groups and in future, serial MECMR studies within the same mouse will also be important.

### Left ventricular function in mouse models of muscular dystrophy cardiomyopathy

We have previously shown that there are differences in function between the *mdx* and *Sgcd−/−* mouse models using the conductance catheter [10], and these results are very consistent despite the very different techniques, though adding information about left ventricular mass and calcium influx. Townsend et al. [24] have compared these mouse models and also found distinct differences (at 6 – 8 months of age). Isolated myocytes from the *mdx* mice show reduced compliance and are susceptible to terminal contracture, whereas the *Sgcd−/−* mice had normal compliance. There was increased fibrosis in the *Sgcd−/−* hearts, though not in the *mdx*. We have also shown that *mdx* mice exhibit improved hemodynamics with 8 weeks of β-blocker treatment though there is significant deterioration in the *Sgcd−/−* mice with reduced heart rate, cardiac output and impaired active relaxation [10]. Despite this information a specific molecular pathway by which these differences occur is still unclear.

### Conclusions and clinical relevance

We have shown at an early stage in the development of muscular dystrophy cardiomyopathy that *in-vivo* calcium influx is increased. Increased calcium influx and left ventricular hypertrophy are seen in both models, though other important haemodynamic and pharmacological differences are apparent, so that all these factors need to be considered together when assessing mechanisms of the cardiomyopathy and response to therapies. A limitation of these *in-vivo* techniques is that it is difficult to say definitively if there are specific abnormalities in channels such as the L-type calcium channel in these models. Thus, the future utility of these non-invasive *in-vivo* techniques will likely be in serially assessing responses to novel preclinical treatments.

## Competing interests

The authors declared that they have no competing interests.

## Authors’ contributions

G: performed scans, analysed data, wrote paper; D: developed MR technique, performed scans, analysed data, contributed to writing paper, B: performed scans, histological studies, contributed to writing paper: L: analysed data, contributed to writing paper, B: developed MR techniques, analysed data, contributed to writing paper, S: conceived study, analysed data, contributed to writing paper; MG: all aspects. All authors read and approved the final manuscript.
